# The Pediatrician

**Published:** 1994

**Authors:** Hoover Adger, Mark J. Werner

**Affiliations:** Hoover Adger, Jr., M.D., M.P.H., is an associate professor in the Department of Pediatrics, Johns Hopkins University School of Medicine, Baltimore, Maryland. Mark J. Werner, M.D., is an assistant professor in the Department of Pediatrics, Vanderbilt University School of Medicine, Nashville, Tennessee

## Abstract

Alcohol consumption by pediatric patients and their parents can have significant impact on the health and development of the children and adolescents. The pediatrician can help prevent or reduce alcohol-induced impairments by providing education and guidance about the responsible use of alcohol and by initiating early intervention if necessary.

Pediatricians deliver health services to patients from birth to 21 years of age. This puts pediatricians in an ideal position to educate children, adolescents, and families about the risks and problems associated with alcohol and other drug use as well as to take an active role in prevention, early identification of affected youth and families, and intervention. Whereas it is relatively easy to identify alcohol-related problems in the most severely affected children and adolescents, the challenge for the pediatrician is to identify the more subtle problems in individuals who are in an early stage of their involvement with alcohol use and to intervene in a timely and meaningful manner. In children affected by parental alcohol abuse, the pediatrician’s task is to recognize the signs of alcohol-induced impairments and initiate appropriate treatment measures.

This article discusses the impact of alcohol use and abuse on the health of infants, children, and adolescents and the alcohol-related problems commonly seen by the pediatrician. It also describes the role of the pediatrician in prevention, screening and in-depth assessment of problem use, brief office-based interventions, and treatment.

## Prevalence of Adolescent Alcohol Use

Alcohol remains the drug of choice for adolescents. In 1992, almost 90 percent of high school seniors reported some experience with alcohol in the past, more than 50 percent reported use in the last month, and 3 percent reported daily use (Johnston et al. 1993). According to the same survey, 23 percent of adolescents often drove after excessive drinking, 17 percent reported problems in peer relationships because of drinking, and 10 percent had been criticized by a close friend for drinking, but only 1 percent believed they had a drinking problem.

Also, there is a trend toward earlier initiation of alcohol use (Johnston et al. 1993). The average age of first drinking alcohol outside of family-sanctioned use or religious occasions is 12 years. Almost 50 percent of the sixth graders surveyed reported feeling peer pressure to try alcohol and 40 percent had drunk beer or wine, yet only 15 percent perceived any risk from drinking alcohol daily (Johnston et al. 1993). In addition, the rate of binge drinking (having five or more drinks in a row) among 10th graders increased from 21 percent in 1992 to 23 percent in 1993. This trend toward drinking at an earlier age is important because the earlier a person begins to drink or use other drugs, the greater the likelihood of related problems later (Yamaguchi and Kandel 1984).

## Impact of Alcohol Use on Children and Adolescents

### Consequences of Adolescent Alcohol Consumption

Alcohol use contributes significantly to accidents, unintended injuries, homicides, and suicides, which are the leading causes of death among teenagers (Rogers and Adger 1993). Approximately 50 percent of fatal motor vehicle crashes and homicides as well as a significant proportion of suicides are associated with alcohol use (National Institute on Alcohol Abuse and Alcoholism [NIAAA] 1990). Post mortem studies show that almost 50 percent of adolescent victims of violent deaths had been drinking alcohol prior to their death (Abel and Zeidenberg 1985; Goodman et al. 1986). Alcohol also has been implicated in a majority of drownings, fire-related deaths, and fatal falls.

Of equal concern is the impact of alcohol use on the cognitive and psychosocial development of young people. Alcohol use, abuse, and dependence contribute significantly to the burden of mental health disorders affecting adolescents (Gans et al. 1990).

Moreover, children and adolescents who drink alcohol often engage in other risk-taking behaviors. There is a correlation between alcohol use and sexual activity for some adolescents, and most date rapes involve alcohol use by one or both partners (*a*U.S. Department of Health and Human Services [USDHHS] 1992*a*). Responsible decisionmaking regarding condom use, partner selection, and sexual abstinence frequently diminishes under alcohol’s influence and contributes to the spread of sexually transmitted diseases, including HIV/AIDS (USDHHS 1992*a*).

### Consequences of Parental Alcohol Consumption

Pediatricians not only see the alcohol-related problems caused by their patients’ drinking but also the problems caused by parental drinking. These include cognitive, emotional, and behavioral problems of children living in households with an alcoholic parent as well as alcohol-related birth defects such as fetal alcohol syndrome (FAS).

#### Children of Alcoholics

Approximately one in eight children in the United States has a parent with a past or current drinking problem (MacDonald and Blume 1986). Even if these children were not directly affected by prenatal alcohol exposure, they may suffer from indirect influences, such as divorce, stress at home, parental anxiety or affective disorders, and frequent changes in family or life situations. Children with alcoholic parents often underestimate their own abilities, which, in combination with actual cognitive deficits or academic problems, can affect their motivation, self-esteem, and academic performance (Bennett et al. 1988). These children also report higher levels of depression and anxiety, exhibit increased symptoms of stress, and generally have more behavioral problems of all types than other children.

Children with alcoholic parents often come to pediatricians with recurring and vague symptoms, such as fatigue, abdominal pain, or musculoskeletal complaints, which are indicative of psychosomatic illness (MacDonald and Blume 1986). In addition, they may suffer from accidental injuries, verbal assault, physical abuse, or incest caused by parental drinking. Pediatricians can help influence these families because of their understanding of family dynamics and close, long-standing relationships with the families.

#### Alcohol-Related Birth Defects

Between 8 and 11 percent of women of childbearing age are either problem drinkers or alcoholics, and approximately 2.6 million infants are born annually following significant intrauterine exposure to alcohol (Wilsnack and Wilsnack 1991). The most notable postnatal effect of significant intrauterine alcohol exposure is FAS. With an incidence estimated at 1.9 cases per 1,000 live births (NIAAA 1993), FAS is the leading known cause of mental retardation in the United States, surpassing Down syndrome.

The term “FAS” refers to a pattern of abnormalities in children of alcohol-abusing mothers. One of the most common manifestations is fetal growth retardation, with weight, length, and head circumference below the 10th percentile for the age group. Also characteristic are facial abnormalities such as small eye openings, a low nasal bridge, an elongated and flattened midface and philtrum (the zone between nose and mouth), and a thin upper lip (NIAAA 1993). The most devastating result of FAS is central nervous system dysfunction, including delayed development, hyperactivity, attention deficits, mental retardation, and seizures.

A long-term followup study of the consequences of FAS illustrated that it is not just a childhood disorder; there are predictable, long-lasting effects with physical, mental, and behavioral implications that persist beyond adolescence into adulthood (Streissguth et al. 1991). Adolescent and adult FAS patients (ages 12 to 40) were found still to have reduced height and head circumference, although the weight deficiency and the characteristic facial features were less marked. Most importantly, the cognitive and developmental handicaps of these FAS patients remained profound, and the majority of patients had maladaptive behaviors such as attention-deficit disorder.

The constellation of features labeled FAS represents the severe end of a continuum of disabilities attributed to maternal alcohol use during pregnancy but does not include all affected individuals. The prevalence of the more subtle consequences, termed “fetal alcohol effects” (FAE), is estimated to be significantly higher (NIAAA 1993). Therefore, although FAS is identified more frequently, the more prevalent alcohol-induced cognitive and behavioral effects of FAE may be the more serious public health problem.

## Risk Factors for Alcohol Use Problems

Biological, psychological, and social risk factors (see box on p. 123) can contribute to the development of alcohol abuse and alcohol-related problems in children and adolescents. For example, genetic predisposition (i.e., a family history of alcoholism) is a well-established biological risk factor. Studies have shown that children of alcoholics are four to five times more likely to develop alcohol dependence than are other children (USDHHS 1992*b*).

**Risk Factors for Alcohol Abuse**

Numerous biological, psychological, and social factors can determine whether an adolescent is at increased risk for developing alcohol abuse patterns and alcohol-related problems.

Family history of alcoholism or other drug abuse
Depression and other psychiatric conditions
Loss of loved one
Low self-esteem
Poor social skills
School problems
Low expectations for school
Family tolerance for deviance
Peer tolerance for deviance

Psychological factors contributing to alcohol-related problems are less well understood. However, children and adolescents with significant problems with behavior (e.g., aggressiveness, rebelliousness, and delinquency), cognition (e.g., learning disabilities and attention-deficit disorders), psychological well-being (e.g., depression, isolation, and low self-esteem), and family functioning (e.g., neglect, abuse, and loss or lack of close relationships) have been shown to be at increased risk for alcohol use problems (Kumpfer 1989; Werner 1991).

Certain family and social influences also have been identified as risk factors for alcohol use disorders or related problems. Kumpfer (1989) and Werner (1991) noted that children whose parents drink alcohol are more likely to drink themselves than are children whose parents do not drink. Other family determinants affecting children’s or adolescents’ drinking behavior include lack of parent-child interactions and maladaptive family problemsolving, which often involve avoidance of issues and conflict. Families with marital discord, financial strains, social isolation, and disrupted family rituals (e.g., meal times, holidays, and vacations) also increase an adolescent’s risk for problem alcohol use.

On the other hand, some family characteristics are considered protective factors against adolescent problem alcohol use. Adolescents least likely to use alcohol and other drugs are emotionally close to their parents, receive advice and guidance from their parents, have siblings who are intolerant of drug use, and are expected to comply with established rules of conduct (Hawkins and Fitzgibbon 1993). The parents of nonusers typically provide praise and encouragement, engender feelings of trust, and are sensitive to their children’s emotional needs.

## Role of the Pediatrician

The Committee on Substance Abuse of the American Academy of Pediatrics (AAP) recommends that pediatricians include substance abuse in their anticipatory guidance (discussed below) to all children and adolescents (Schuydower et al. 1993). To fulfill this role adequately, pediatricians must develop interviewing and counseling skills to recognize risk factors and signs of substance abuse in preadolescent and adolescent patients. In addition, they must learn to perform an individualized assessment of “normal” versus “problematic” behavior and to initiate appropriate interventions or referral.

The Guidelines for Adolescent Preventive Services (GAPS), established by the American Medical Association, recommends both primary (e.g., patient education and anticipatory guidance) and secondary (e.g., early intervention) prevention strategies to reduce adolescent use of alcohol and other drugs, including steroids (Elster and Kuznets 1994). These measures include screening all children and adolescents and using brief counseling interventions and referral as needed. GAPS also recommends that pediatricians routinely ascertain their patients’ risk factors (including a family history of alcoholism) in the medical history and conduct screening evaluations for all school-aged children and adolescents.

### Anticipatory Guidance and Prevention

The role of the pediatrician in preventing alcohol-related problems begins early in a child’s life, ideally during neonatal visits, by educating the parents about their responsibilities and the influence of their lifestyle and alcohol use on the infant, child, and adolescent and by exploring their attitude toward alcohol use. Parents need to be aware that their attitudes and beliefs can strongly influence their child’s behavior.

Attitudes and beliefs regarding alcohol develop early in life, often by age 7 or 8 (Gaines and Brooks 1988). Therefore, well-child visits during the early school years allow the pediatrician to begin anticipatory guidance and to talk with children and parents about alcohol use. Pediatricians can initiate or enhance the dialog between children and their parents by asking if alcohol use is discussed in school, inquiring about the specifics of what is being taught, and assessing if the child understands the messages that are being delivered. It is important to ask if alcohol use is discussed among friends, whether alcohol is present in the child’s environment, and about the child’s perceptions of why some people drink and whether alcohol use is harmful.

In addition to providing anticipatory guidance to individual patients, pediatricians can play an active role in general prevention programs directed at children and adolescents. Pediatricians can act as important advocates for appropriate community- and school-based prevention approaches (Comerci and MacDonald 1990), ensuring that local programs are culturally relevant and appropriate for the community and population they serve.

Some of the most frequently used general prevention approaches in community-and school-based programs focus on deterring initial alcohol and other drug use (Comerci and MacDonald 1990; Hawkins and Fitzgibbon 1993). Although their content may vary, these approaches encompass problemsolving, decisionmaking, interpersonal skills, assertiveness training, cognitive skills for resisting social pressures, and drug-free coping alternatives. These skills are taught through demonstration, rehearsal, or homework assignments.

### Recognition of Alcohol-Related Problems

Pediatric patients show different degrees of participation with alcohol, from contemplating or experimenting with alcohol use to being involved with alcohol to a harmful degree or alcohol dependent (MacDonald 1984; Adger 1991). The challenge for the pediatrician is to recognize and, if necessary, counteract these different stages of alcohol use and abuse.

The signs and symptoms of alcohol abuse in adolescents often are subtle, indicated only by behavioral dysfunctions. Adolescents at a stage of initial or experimental use may not yet display any behavioral changes or discernible consequences. But alcohol-related symptoms and associated consequences, such as falling grades; a sudden lapse in school attendance; and problems with interpersonal relationships, family, or the law may become evident as alcohol use increases. Other symptoms include weight loss, change in sleep habits and energy level, depressed mood or mood swings, and suicidal thoughts or suicide attempts. Because of this wide variety of symptoms, the pediatrician must consider alcohol use as a potential cause for all behavioral, family, psychosocial, or related medical problems.

#### Routine Screening

Pediatricians should screen all patients for alcohol use and determine the need for further assessment and intervention. Potential risk factors and behaviors should be reviewed with the patients and their parents as a routine part of each pediatric visit. Pediatricians also should address environmental stressors, social pressures, and family attitudes and practices, which play an important role in shaping the attitudes and behaviors of young people.

Although there is no broad acceptance of any one screening strategy in a pediatric setting, several approaches to office screening and assessment of alcohol and other drug use are available and offer guidance for the pediatrician (Anglin 1987; Bailey 1989; Farrow and Deisher 1986; Alderman et al. 1992; see this page for guidelines for the screening interview).

**Guidelines for the Screening Interview**

**Figure:**
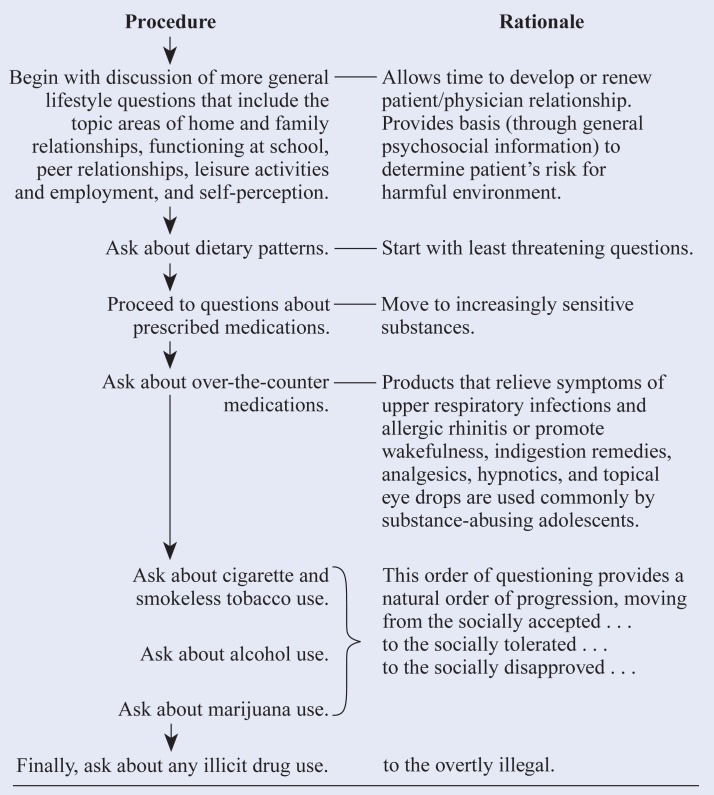

SOURCE: Adapted from Anglin 1987.

Several authors recommend using the CAGE test (see Nilssen and Cone, pp. 136–139), a widely used screening questionnaire that can be modified to meet the needs of the adult or pediatric patient (e.g., MacDonald 1986). The four CAGE questions are short and simple and can be incorporated easily into taking the medical history.

In addition to being useful for screening an adolescent’s alcohol use directly, the CAGE questions also can provide a proxy report regarding another person. They can be adapted to reveal the perceptions of others about the adolescent’s use of alcohol or the alcohol use of a parent or significant other. For example, the pediatrician could use the CAGE questions in the following manner with a child or adolescent who is not using alcohol but seems concerned about a parent’s use of alcohol:

- Do you think your parent needs to Cut down on his/her alcohol use?
- Does your parent get Annoyed at comments about his/her drinking?
- Does your parent ever feel Guilty about his/her drinking?
- Does your parent ever take a drink early in the morning as an Eye opener?

Several other questionnaires can be used for screening adolescents, such as the Drug and Alcohol Problem Quick Screen, Adolescent Alcohol Involvement Scale (AAIS), Personal Experience Screening Questionnaire (PESQ), Children of Alcoholics Screening Test (CAST), and the Substance Abuse Subtle Screening Inventory (SASSI). Another instrument that helps to evaluate multiple problem areas and can be adapted easily to the office setting is the Problem Oriented Screening Instrument for Teenagers (POSIT) (for an overview, see Comerci 1993).

The use of specific interviewing techniques and questionnaires to obtain information about adolescent alcohol consumption remains an area of active discussion. Whatever approach is used, the acquisition of accurate and meaningful information from the child or adolescent will depend largely on the degree to which trust is established and the patient perceives the pediatrician as caring, empathetic, and knowledgeable.

#### Assessment

Screening interviews are only an important and time-saving first step to identifying an alcohol problem. A positive screening result indicates the need for an in-depth assessment and a formal diagnosis. Assessment is a more lengthy and structured process designed to determine the extent of the problem, explore coexisting medical and psychiatric conditions, and assist in treatment planning (see box on p. 125).

**Differences Between Screening and Assessment Interviews**

**Table:** 

	Screening	Assessment
		
**Goal**	Determine *existence* of problem	Determine *extent* of problem, explore comorbidities, assist in treatment planning
**Target Population**	All patients (including patients’ family members)	Patients with positive screening results
**Administered by**	Any health care provider	Specially trained personnel
**Administration Time Range**	5–20 minutes	45 minutes to 2–3 hours
**Approaches/Tools**	CAGE, CAGE-AID Problem Oriented Screening Instrument for Teenagers (POSIT) Drug and Alcohol Problem (DAP) Quick Screen Adolescent Alcohol Involvement Scale (AAIS) Relax, Alone, Friends, Family, Trouble (RAFFT) MAST adapted for adolescents Personal Experience Screening Questionnaire (PESQ) Alcohol Use Disorders Identification Test (AUDIT) Perceived Benefits of Drinking and Drug Use Children of Alcoholics Screening Test (CAST)	Personal Experience Inventory (PEI) Adolescent Diagnostic Interview (ADI) Adolescent Assessment/Referral System (AARS)

SOURCE: Adapted from *The Johns Hopkins Pediatric Substance Abuse Curriculum Manual*, 1994.

A comprehensive assessment requires information about the physiological, psychological, behavioral, and social aspects of the patient’s life. Because of this broad scope, assessment and diagnosis may be beyond the time limitations and skills of most practitioners. The pediatricians may therefore choose to make a referral to a skilled alcohol abuse specialist.

### Brief Office-Based Interventions

Early intervention is directed at patients whose use of alcohol, tobacco, or other drugs places them at an unacceptably high risk for negative consequences or has resulted in clinically significant dysfunctions or consequences and at patients or families who exhibit specific problem behaviors considered to be precursors to alcohol problems (Klitzner et al. 1992). The rationale for these interventions is that health messages provided by pediatricians can effect behavioral changes in adolescents because the physician is considered a source of credible information. These messages can be especially effective if they reinforce information already received in other settings from teachers, parents, and other adults (Pentz 1993). Personalized interactions with the adolescent in an office setting also may have a greater impact than preventive interventions provided in group settings or through mass media.

Interventions for alcohol problems should provide adolescents with the necessary information, skills, and support to change their behavior. The primary impact of brief interventions is motivational—triggering a decision and commitment to change. Brief interventions typically have three common elements.

First, after an initial evaluation, the patient is given structured feedback about the screening results. This provides the patient with an opportunity to reflect in detail on his or her present situation.

Second, the pediatrician clearly advises the patient to make a change toward a specific goal, such as total abstinence, elimination of hazardous use, or entering treatment. The patient also can be presented with a menu of alternative strategies for changing drinking behavior. Although pediatricians should support the primary objective of abstinence by adolescents under the age of 21, the most urgent message—in terms of both immediate and lifetime behaviors to reduce morbidity and mortality—is to not drink and drive or ride with others who drink and drive (Beach 1991).

Third, the patient’s responsibility for change is emphasized, often through explicit messages, for example, “It’s up to you to decide what to do with this situation. Nobody can decide for you, and no one can change your drinking if you don’t want to change.”

The essential office-based interventions for alcohol problems include the following:

- Ask older children and adolescents about their awareness of and use of alcohol.
- Ask about the use of alcohol by the patient’s friends.
- Ask if the adolescent has ever driven while under the influence of alcohol or ridden with someone who was under the influence.
- Have the adolescent commit to a firm no-drinking-and-driving policy.
- Help families develop safe-ride policies before they are needed.
- Screen for alcohol use problems as part of the evaluation of all patients who sustained accidental trauma or were in motor vehicle crashes.
- Identify and refer substance-abusing patients or family members.

Not only the kind of advice but also the way in which it is delivered is important. Effective brief interventions emphasize the supportive nature of the physician-patient relationship and seek to reinforce the patient’s self-efficacy or optimism. Overly directive and confrontational styles tend to evoke high levels of patient resistance, whereas a more empathetic style is associated with less resistance and better long-term change (Miller and Rollnick 1991).

### Treatment

The role of the pediatrician in treating alcohol-related problems varies considerably. A significant function is to assist the patients and their families in selecting the appropriate treatment program. Several treatment alternatives exist for alcohol-dependent children and adolescents, including short-term inpatient treatment; residential programs; and outpatient care, which spans the spectrum from office-based care to intensive and structured day programs (AAP 1990).

It is important for the pediatrician to be aware of the different treatment programs and resources in the community and their particular treatment philosophies. Although most treatment programs begin with the interruption of alcohol use, require continued abstinence from alcohol, and have the goal of a drug-free lifestyle, there is a wide variety of settings and several approaches to treating children and adolescents.The AAP (1990) has issued guidelines for evaluating substance abuse treatment programs.

There has been little treatment outcome research evaluating the impact or therapeutic benefit of particular treatment approaches in adolescents. According to a recent Institute of Medicine report, the state of knowledge about adolescent treatment is less than satisfactory. The number of studies on adolescents is small, and most work is based on older treatment models. Hence, there is a need for studies that specifically address assessment of alcohol-related problems in children and adolescents and matching of appropriate treatment with the level of involvement and severity of consequences.

## Conclusion

Pediatricians encounter children, adolescents, and families suffering from alcohol-related problems of various kinds and intensities. The challenge for the physician is to detect patients at risk for or in early stages of alcohol abuse. Therefore, pediatricians must be acutely aware of the many avenues by which alcohol problems affecting children and adolescents present themselves. By including routine screening tests in their practice and initiating appropriate interventions or referrals, pediatricians can make a difference in the long-term health and development of their patients.

## References

[b1-arhw-18-2-121] Abel EL, Zeidenberg P (1985). Age, alcohol and violent death: A postmortem study. Journal of Studies on Alcohol.

[b2-arhw-18-2-121] Adger H (1991). Problems of alcohol and other drug use and abuse in adolescents. Journal of Adolescent Health.

[b3-arhw-18-2-121] Alderman EM, Schonberg SK, Cohen MI (1992). The pediatrician’s role in the diagnosis and treatment of substance abuse. Pediatrics in Review.

[b4-arhw-18-2-121] American Academy of Pediatrics Provisional Committee on Substance Abuse (1990). Selection of substance abuse treatment programs. Pediatrics.

[b5-arhw-18-2-121] Anglin TM (1987). Interviewing guidelines for the clinical evaluation of adolescent substance abuse. Pediatric Clinics of North America.

[b6-arhw-18-2-121] Bailey GW (1989). Current perspectives on substance abuse in youth. Journal of the American Academy of Child and Adolescent Psychiatry.

[b7-arhw-18-2-121] Beach RK (1991). Prioritizing health behaviors in adolescents: Health promotion in the clinical setting. Adolescent Health Update.

[b8-arhw-18-2-121] Bennett LA, Wolin SJ, Reiss D (1988). Cognitive, behavioral and emotional problems among school-age children of alcoholics. American Journal of Psychiatry.

[b9-arhw-18-2-121] Comerci CG (1993). Office assessment of substance abuse and addiction. Adolescent Medicine: State of the Art Reviews.

[b10-arhw-18-2-121] Comerci CG, Macdonald DI (1990). Prevention of substance abuse in children and adolescents. Adolescent Medicine: State of the Art Reviews.

[b11-arhw-18-2-121] Elster AB, Kuznets NJ (1994). AMA Guidelines for Adolescent Preventive Service (GAPS).

[b12-arhw-18-2-121] Farrow JA, Deisher R (1986). A practical guide to the office assessment of substance abuse. Pediatric Annals.

[b13-arhw-18-2-121] Gaines LS, Brooks P (1988). Children’s knowledge of alcohol and the role of drinking. Journal of Applied and Developmental Psychology.

[b14-arhw-18-2-121] Gans JE, Blyth DA, Elster AB, Gaveras LL (1990). America’s adolescents: How healthy are they?. AMA Profiles of Adolescent Health.

[b15-arhw-18-2-121] Goodman RA, Mercy JA, Loya F, Rosenberg ML, Smith JC, Allen NH, Vargas L, Kolts R (1986). Alcohol use in interpersonal violence: Alcohol detected in homicide victims. American Journal of Public Health.

[b16-arhw-18-2-121] Hawkins DJ, Fitzgibbon JJ (1993). Risk factors and risk behaviors in prevention of adolescent substance abuse. Adolescent Medicine: State of the Art Reviews.

[b17-arhw-18-2-121] Johnston LD, O’Malley PM, Bachman JG (1993). National Survey Results on Drug Use From Monitoring the Future Study, 1975–1992. Volume 1: Secondary School Students.

[b18-arhw-18-2-121] Klitzner M, Fisher D, Stelart K, Gilbert S (1992). Substance Abuse: Early Interventions for Adolescents.

[b19-arhw-18-2-121] Kumpfer KL (1989). Prevention of alcohol and drug abuse: A critical review of risk factors and prevention strategies. Prevention of Mental Disorders, Alcohol, and Other Drug Use in Children and Adolescents. OSAP Prevention Monograph–2.

[b20-arhw-18-2-121] MacDonald DI (1984). Drugs, drinking and adolescence. American Journal of the Diseases of Children.

[b21-arhw-18-2-121] MacDonald DI (1986). How you can help prevent teenage alcoholism. Contemporary Pediatrics.

[b22-arhw-18-2-121] MacDonald DI, Blume SB (1986). Children of alcoholics. American Journal of Diseases of Children.

[b23-arhw-18-2-121] Miller WR, Rollnick S (1991). Motivational Interviewing: Preparing People to Change Addictive Behavior.

[b24-arhw-18-2-121] National Institute on Alcohol Abuse and Alcoholism (1990). Seventh Special Report to the U.S. Congress on Alcohol and Health.

[b25-arhw-18-2-121] National Institute on Alcohol Abuse and Alcoholism (1993). Eighth Special Report to the U.S. Congress on Alcohol and Health.

[b26-arhw-18-2-121] Pentz MA, Elster AD, Panzerine S, Holt K (1993). Benefits of integrating strategies in different settings. AMA State of the Art Conference on Adolescent Health Promotion: Proceedings.

[b27-arhw-18-2-121] Rogers PD, Adger H (1993). Alcohol and adolescents. Adolescent Medicine: State of the Art Reviews.

[b28-arhw-18-2-121] Schuydower M, Fuller PG, Heyman RB, Jacobs EA, Pruitt AW, Sutton JM, Tenenbein M (1993). Role of the pediatrician in prevention and management of substance abuse. Pediatrics.

[b29-arhw-18-2-121] Streissguth AP, Aase JM, Clarren SK, Randels SP, LaDue RA, Smith DF (1991). Fetal alcohol syndrome in adolescents and adults. Journal of the American Medical Association.

[b30-arhw-18-2-121] U.S. Department of Health and Human Services, Office of the Inspector General (1992a). Youth and Alcohol: Dangerous and Deadly Consequences.

[b31-arhw-18-2-121] U.S. Department of Health and Human Services (1992b). Alcoholism Tends to Run in Families.

[b32-arhw-18-2-121] Werner MJ (1991). Adolescent Substance Abuse: Risk Factors and Prevention Strategies.

[b33-arhw-18-2-121] Wilsnack SC, Wilsnack RW (1991). Epidemiology of women’s drinking. Journal of Substance Abuse.

[b34-arhw-18-2-121] Yamaguchi K, Kandel DB (1984). Patterns of drug use from adolescence to young adulthood: III. Predictors of progression. American Journal of Pediatric Health.

